# Physiopathological Role of Neuroactive Steroids in the Peripheral Nervous System

**DOI:** 10.3390/ijms21239000

**Published:** 2020-11-26

**Authors:** Eva Falvo, Silvia Diviccaro, Roberto Cosimo Melcangi, Silvia Giatti

**Affiliations:** Dipartimento di Scienze Farmacologiche e Biomolecolari, Università degli Studi di Milano, 20133 Milan, Italy; eva.falvo@unimi.it (E.F.); silvia.diviccaro@gmail.com (S.D.); silvia.giatti@unimi.it (S.G.)

**Keywords:** peripheral neuropathy, pain, diabetes mellitus, physical injury, chemotherapy-induced peripheral neuropathy, steroidogenesis, progesterone, testosterone, sex difference

## Abstract

Peripheral neuropathy (PN) refers to many conditions involving damage to the peripheral nervous system (PNS). Usually, PN causes weakness, numbness and pain and is the result of traumatic injuries, infections, metabolic problems, inherited causes, or exposure to chemicals. Despite the high prevalence of PN, available treatments are still unsatisfactory. Neuroactive steroids (i.e., steroid hormones synthesized by peripheral glands as well as steroids directly synthesized in the nervous system) represent important physiological regulators of PNS functionality. Data obtained so far and here discussed, indeed show that in several experimental models of PN the levels of neuroactive steroids are affected by the pathology and that treatment with these molecules is able to exert protective effects on several PN features, including neuropathic pain. Of note, the observations that neuroactive steroid levels are sexually dimorphic not only in physiological status but also in PN, associated with the finding that PN show sex dimorphic manifestations, may suggest the possibility of a sex specific therapy based on neuroactive steroids.

## 1. Introduction

Peripheral neuropathy (PN) is one of the most common disorders affecting the function of one (i.e., mononeuropathy) or more peripheral nerves (i.e., multi- or polyneuropathy), with a prevalence of about 2.4% in the general population that reaches 8% with aging [1]. This pathological condition may be either inherited or acquired. Inherited forms of PN are a group of diseases altogether referred to as Charcot–Marie–Tooth disease, including demyelinating and axonal variants [2]. Instead, acquired peripheral neuropathy may appear during the aging process, in infections, autoimmune disorders (e.g., AIDS, rheumatoid arthritis, Guillain–Barrè syndrome, sarcoidosis, systemic lupus erythematosus), in systemic or metabolic disorders (e.g., diabetes mellitus, kidney failure, vitamin deficiencies, alcoholism), after exposure to toxic compounds (e.g., nitrous oxide, mercury, organic solvents) and during drug treatment (e.g., chemotherapeutics, anti-tuberculosis medication, antiretroviral). The pathological reaction of peripheral nerves to these various insults results in a degeneration of the nerve fibers that may occur as axonal damage and/or primary demyelination. The most common condition is axonal degeneration, that occurs in metabolic deregulation, ischemia, toxin exposure and inherited disorders [3].

Patients affected by PN may present, in term of form and intensity, very different clinical manifestations, such as motor insufficiency (weakness and muscle waste), sensory abnormalities (numbness, pain, hyperalgesia/allodynia and paresthesia), autonomic symptoms (blurred vision, altered sweating, nausea or vomiting after meal, urinary hesitancy, male impotence, decreased ability to regulate body temperature, orthostatic hypotension) or a combinations of all, often depending on the particular disorder [3].

Diagnosis of PN involves a neurophysiological examination of muscles and peripheral nerves as well as patient complete history to determine the possible etiology. However, this remains unknown in approximately 50% of cases [4], especially for most of the axonal neuropathies. In contrast, clinical evidence of a demyelinating neuropathy increases the possibility of delineating the etiology and starting an effective treatment with immune-modulatory drugs [4]. 

A possible strategy to find diagnostic markers as well an effective pharmacological treatment for PN could be to shift the focus to new biological targets such as those that are involved in relevant molecular events in the peripheral nervous system (PNS). From this perspective, neuroactive steroids may represent a promising option. These molecules are able to modulate nervous system functions. They can originate from peripheral steroidogenic tissues, like gonads and adrenal glands, or from the nervous system itself, and for this reason they are also called neurosteroids [5]. Different studies have reported that peripheral nerves are able to synthesize and metabolize these molecules and express their receptors [6]. Therefore, as will be here discussed, peripheral nerves are a target for neuroactive steroid action. Indeed, they are involved in the regulation of different functions of peripheral nerves, such as myelination and Schwann cell proliferation [7,8,9,10,11,12,13,14,15,16,17,18,19,20,21,22,23,24,25,26].

## 2. Synthesis, Metabolism, and Receptors of Neuroactive Steroids in the Peripheral Nervous System

**Figure 1:**
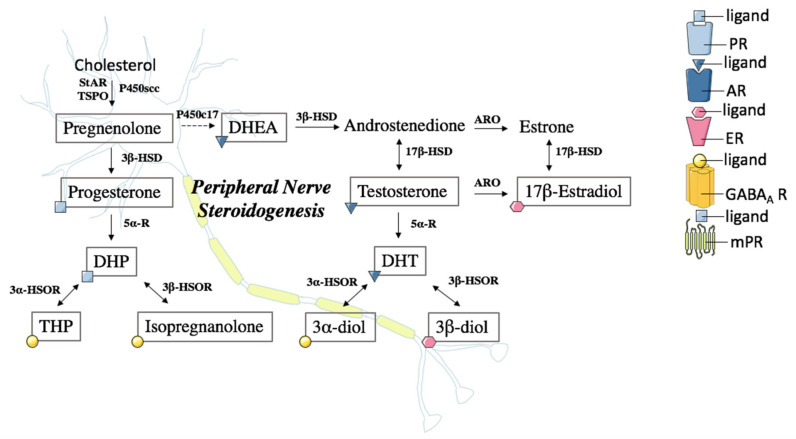
Steroidogenesis in the peripheral nervous system. Schematic representation of steroidogenic pathways. The arrows and bidirectional arrows indicate the irreversible and reversible reactions, respectively. The dashed line indicates a non-direct conversion. Steroids in squares represent molecules assessed by liquid chromatography tandem mass spectrometry in rat sciatic nerve. Further details are explained in the text. 3α-diol: 5α-androstane-3α,17β-diol; 3α-HSOR: 3α- hydroxysteroid oxidoreductase; 3β-diol: 5α-androstane-3β,17β-diol; 3β-HSD: 3β-hydroxysteroid dehydrogenase; 3β-HSOR: 3β-hydroxysteroid oxidoreductase; 5α-R: 5α-reductase; 17β-HSD: 17β-hydroxysteroid dehydrogenase; AR: Androgen receptor; ARO: Aromatase; DHEA: Dehydroepiandrosterone; DHP: Dihydroprogesterone; DHT: Dihydrotestosterone; ER: estrogen receptor; GABA_A_ R: γ-amino butyric acid type A receptor; mPR: membrane progesterone receptor; P450scc: cytochrome P450 side chain cleavage; PR: progesterone receptor; StAR: Steroidogenic Acute Regulatory Protein; THP: Tetrahydroprogesterone; TSPO: Translocator protein-18kDa.

PNS is a source of neuroactive steroids, as reported in Figure 1 [5,6,27,28]. The first step of neurosteroidogenesis is the formation of pregnenolone (PREG) from cholesterol by the action of cytochrome P450 side chain cleavage (P450scc). This step requires the transport of cholesterol from intracellular stores to the inner mitochondrial membrane by the support of a molecular complex that includes several proteins, such as the translocator protein-18kDa (TSPO) and the steroidogenic acute regulatory protein. These proteins, together with cytochrome P450scc, are expressed by Schwann cells and the neuronal compartment in dorsal root ganglia (DRG) [29,30,31]. Indeed, these cells are also able to convert PREG into other neuroactive steroids. For example, they express steroidogenic enzymes, such as 3β-hydroxysteroid dehydrogenase (3β-HSD), which converts PREG into progesterone (PROG), 5α-reductase (5α-R) and 3α- or 3β-hydroxysteroid oxidoreductase (3α-HSOR or 3β-HSOR), converting PROG and testosterone (T) into their 5α- and 3α- or 3β-hydroxy-5α-reduced metabolites [6,32,33]. In particular, PROG is converted into dihydroprogesterone (DHP) and subsequently into tetrahydroprogesterone (THP), also known as allopregnanolone, or into isopregnanolone (i.e., the 3β isomer of THP). Similarly, T is converted into dihydrotestosterone (DHT) and then into 5α-androstane-3α,17β-diol (3α-diol) or 5α-androstane-3β,17β-diol (3β-diol) [32]. T may be also metabolized to 17β-estradiol by the enzyme aromatase, even if the main pathway for 17β-estradiol synthesis in endocrine tissues is via androstenedione and estrone conversion [34]. The functional presence of these enzymes has been also confirmed by liquid chromatography tandem mass spectrometry assessment of neuroactive steroid levels in the peripheral nerves (Figure 1). Indeed, levels of PREG, PROG and its metabolites (i.e., DHP, THP and isopregnanolone), T and its metabolites (i.e., DHT, 3α-diol and 3β-diol) and dehydroepiandrosterone (DHEA), a neuroactive steroid obtained from PREG by the action of cytochrome P450c17, have been reported in the rat sciatic nerve. Interestingly, the levels observed in the nervous system do not reflect exactly what is detected in plasma, further confirming the production and metabolism of these neuroactive steroids in PNS [35,36,37,38]. 

In addition to be able to synthesize and metabolize neuroactive steroids, peripheral nerves and Schwann cells also express classical and non-classical steroid receptors; thus, they represent a target for the actions of neuroactive steroids (Figure 1). Classical steroid receptors, such as PROG (PR), androgen (AR), estrogen, glucocorticoid and mineralocorticoid receptors, that bind PROG, DHP, T, DHT, DHEA, estrogens, glucocorticoid and mineralocorticoid, have been detected in neuronal compartment of PNS (i.e., DRG) and in glia (i.e., Schwann cells) [6,39,40,41,42,43,44,45,46]. In addition, some neuroactive steroid metabolites such as 3α,5α-reduced neuroactive steroids, are known to also bind neurotransmitter receptor channels and/or to modulate their expression. For example, N-methyl-D-aspartate (NMDA), γ-amino butyric acid type A and B (GABA_A_ receptor, GABA_B_ receptor), serotonin type 3 (5-HT_3_) and α-amino-3-hydroxy-5-methyl-4-isoxazolepropionic acid (AMPA) [47,48,49,50,51,52,53,54]. In particular, GABA_A_ (i.e., α2, α3, β1, β2 and β3 subunits) and GABA_B_ (i.e., GABA_B1_ and GABA_B2_) receptors have been identified in peripheral nerves and Schwann cells [15,20,55]. Furthermore, the rat sural nerve expresses AMPA subunits, NMDA receptor 1 subunits, GluR 5,6 and 7 kainate subunits [56,57], and Schwann cells of mammalian peripheral vestibular system express GluR 2,3 and 4 [57,58]. Finally, PROG and its metabolites may also bind the membrane progesterone receptors (mPRs) [59,60]. All five mPRs isoforms as well as progesterone receptor membrane component 1 (PGRMC1) have been detected in Schwann cells [61,62] (Figure 1).

## 3. Physiological Effects of Neuroactive Steroids in the Peripheral Nervous System

One of the best characterized actions of neuroactive steroids in the PNS is the regulation of the myelin program. Namely, neuroactive steroids exert their physiological actions on the expression of myelin proteins as well as of transcription factors involved in the regulation of myelination acting on classical and non-classical steroid receptors. Indeed, PROG and its metabolites modulate the expression of myelin proteins of the PNS, such as myelin basic protein (MBP), myelin proteolipid protein, glycoprotein zero (P0) and peripheral myelin protein (PMP22) as well as myelin formation [6,8,9,20,63,64]. In particular, the expression of P0 in the sciatic nerve of adult male rats, as well as that in Schwann cell culture, is increased by treatment with PROG, DHP or THP. On the contrary, in the case of PMP22, only THP is effective [6,20]. Neuroactive metabolites of T exert similar effects on the synthesis of myelin proteins. Indeed, in adult male rats, orchidectomy decreases the expression of both P0 and PMP22 in the sciatic nerve, and further treatment with DHT or 3α-diol is able to restore the levels of P0 and PMP22, respectively [14,65]. A very similar effect is also evident in cultures of rat Schwann cells; treatment with DHT increases levels of P0 mRNA [14], while 3α-diol increases PMP22 expression [21]. The mechanism underlying these different effects seems to be dependent on the myelin proteins considered. Indeed, it has been observed that the expression of P0 is under the control of classical steroid receptors, like PR and AR, while a role for non-classical receptors, such as the GABA_A_ receptor, has been hypothesized in the case of PMP22 [6]. In particular, the role of PR in regulating P0 expression has been confirmed by in vitro and in vivo experiments. For instance, in rat Schwann cell cultures, mifepristone, an antagonist of PR, is able to block the stimulatory effect exerted by PROG or its reduced metabolite, DHP. In agreement, in vivo treatment with mifepristone from the first day of life reduces in 20 days the expression of P0 in the sciatic nerve of rats [66]. In addition, it has been demonstrated that a coactivator, the steroid receptor coactivator-1 (SRC-1), participates in the effect of DHP on P0 gene expression. Finally, as demonstrated in an immortalized Schwann cell line (i.e., MSC80 cells), the stable transfection, in order to over- or down-express SRC-1, produces, after DHP treatment, an increase or a complete loss of P0 expression, respectively [67]. In further support of PR functioning by nuclear receptor coactivators in PNS, it has been demonstrated that in female Schwann cells, PR is co-expressed with another member of the p160 family, such as SRC-2 [68]. 

Similarly, a role for AR in controlling expression of P0 has also been hypothesized. Indeed, in vivo chronic treatment with flutamide, an antagonist of this steroid receptor, decreases the synthesis of P0 in the sciatic nerve of rats [65]. Interestingly, inhibition of AR activity influences synthesis of P0 at adult age only. Therefore, altogether these observations suggest that PROG and its metabolites are important for inducing P0 synthesis during the first steps of the myelination process, while T and its metabolites subsequently participate in the maintenance of this process. The role of classical steroid receptors in controlling P0 expression is further supported by the observation that putative progesterone responsive elements as well as androgen responsive elements have been reported on the P0 gene [14]. This finding further supports the concept that both PROG and androgens may control P0 expression.

In contrast to what was observed on P0, the regulation of PMP22 expression appears to be under the control of the GABA_A_ receptor. For instance, treatment with a GABA_A_ receptor antagonist, such as bicuculline, completely abolishes the stimulatory effect exerted by THP on PMP22, while treatment with an agonist, such as muscimol, exerts a stimulatory effect on PMP22, which is comparable to that exerted by THP [69].

Neuroactive steroids, in addition to regulating the expression of myelin proteins, also have an action on transcription factors that play an important role in the physiology of Schwann cells and in their myelinating program. Indeed, it has been shown that in cultures of Schwann cells PROG stimulates the gene expression of Krox-20, Krox-24, Egr-3 and FosB [12,23]. In addition, PROG metabolites affect the synthesis of transcription factors. For instance, Krox-20 is stimulated by treatment with DHP or THP, while Sox-10 is only stimulated by DHP [16].

In addition to the consolidated capability of THP in controlling myelination in PNS, a recent study has demonstrated that this neuroactive steroid is also important in regulating the development and maturation of Schwann cells. These effects are mediated mostly by GABA_A_ receptor and Src/FAK (i.e., non-receptor-type tyrosine kinases involved in the regulation of Schwann cells proliferation, survival and differentiation) signaling cascade activation, causing actin remodeling, migration and proliferation of Schwann cells [70]. 

Not only the metabolites, but also PROG itself, affect Schwann cell proliferation. For example, as demonstrated by in vitro experiments, PROG has a stimulatory effect on proliferation of these peripheral glial cells [25,71]. A recent study has shown that mPRs also play a potential role in the PNS physiology, and specifically in Schwann cells. Indeed, activation of mPRs modulates the expression of the myelin-associated glycoprotein as well as of several Schwann cell differentiation markers; moreover, it induces morphological changes and increases Schwann cell migration and proliferation [61,62]. 

Finally, androgens also have an effect on Schwann cell proliferation. For instance, the number of terminal Schwann cells unsheathing the synaptic junction between muscles and motor nerve endings decreased after castration and this effect is neutralized by T administration [13].

## 4. Protective Effects of Neuroactive Steroids in the Peripheral Nervous System

Neuroactive steroids, beside having protective effects in the central nervous system (CNS) [72,73,74,75,76,77,78,79,80,81,82,83,84,85,86], are also effective in PN [17,21,22,68,85,87,88,89,90,91,92,93]. As mentioned above, neuroactive steroids may influence many parameters of PNS and, for this reason, the potential effectiveness of these molecules has been tested in several experimental models of PN. A background for possible protective effects of neuroactive steroids was also provided by the finding that the levels of neuroactive steroids are affected in many types of PN [36,37,38,84,89,94,95,96].

### 4.1. Diabetic Peripheral Neuropathy

More than 50% of patients affected by diabetes mellitus show impairment of the PNS. Different types of abnormalities can be seen, such as mononeuropathy, mononeuritis multiplex, and polyneuropathy. In particular, this last condition is characterized by structural and functional alteration of the PNS, such as nerve conduction velocity (NCV) reduction, paranodal demyelination and degeneration of axons [97,98]. Diabetic patients as well as experimental models of diabetes, such as streptozotocin (STZ)-treated rats (i.e., an experimental model of type 1 diabetes), show reduced activity of the Na^+^,K^+^-ATPase enzyme and altered nociception, due to a decrease in the number of sensitive fibers [99,100,101,102,103,104,105,106,107]. In the same experimental model, the gene expression of P0 and PMP22 has been demonstrated to be affected by the pathology [101,106]. Interestingly, infoldings and outfoldings, representing myelin invaginations and evaginations in peripheral myelinated nerves respectively, have been observed. Moreover, other morphological alterations, like altered compaction of myelin, have been reported [108]. In particular, diabetes seems to alter phospholipids, fatty acids and cholesterol content in the myelin of the sciatic nerve, leading to membrane fluidity modification [109]. The experimental model of STZ, three months of diabetes, also induces, in the sciatic nerve, a decrease of neuroactive steroid levels, such as PREG, PROG, T and their metabolites (i.e., DHT, 3α-diol and THP) [38]. In addition, a decrease in neuroactive steroid levels associated with a disruption of axonal transport and alteration of mitochondrial function has been also reported after one month of diabetes (i.e., short-term diabetes) [110].

As extensively demonstrated, neuroactive steroids show important neuroprotective functions in STZ-rats. Indeed, PROG and DHP are able to prevent the increase in the number of fibers with myelin infoldings in the sciatic nerve of STZ-rats [108]. Neuroactive steroids may also influence biochemical and functional parameters of peripheral nerves. For instance, DHEA prevents vascular and neuronal dysfunction in the sciatic nerve of diabetic rats [111]. In addition, PROG and its metabolites, DHP and THP, improve the impairment of NCV and the activity of Na^+^, K^+^-ATPase, and restore skin innervation density and the altered level of PMP22 and P0 [101]. An effect of DHP has also been reproduced in an ex vivo model of hyperglycemia. Thus, DRG cultures exposed to high levels of glucose show reduced immunostaining for MBP and DHP is able to counteract this effect [89]. In the experimental model, alterations of lipid metabolism have also been observed. DHP treatment of STZ-rats was able to improve lipid content in peripheral myelin membranes, fatty acid desaturation, and sterol regulatory element binding protein (SREBP)-1c expression, thus promoting alteration of myelin structure [112]. Cross-talk between fatty acid biosynthesis and neuroactive steroids is also supported by observations performed in SREBP-1c knock-out male mice. Indeed, this experimental model shows PN features [113] associated with altered levels of PREG, PROG, T and their reduced metabolites [96].

In addition, T and its metabolites exert neuroprotective effects in diabetic peripheral neuropathy. Indeed, DHT or 3α-diol treatment improves NCV and counteracts the increase of thermal threshold induced by diabetes, while only DHT treatment improves the activity of Na^+^, K^+^-ATPase and the expression of P0 in the sciatic nerve. T, DHT and 3α-diol also reverse the reduction of intra-epidermal nerve fiber density induced by diabetes [106]. In this experimental model of PN, a neuroprotective role of the Liver X Receptor (LXR) has been also demonstrated [114]. Indeed, activation of LXR with GW3965 (i.e., a synthetic ligand of LXR), by promoting steroidogenesis, exerts protective effects on altered thermal nociception, NCV and Na^+^, K^+^-ATPase activity [114]. Similarly, TSPO ligands reduce the severity of diabetic neuropathy. Indeed, it has been demonstrated that treatment with Ro5-4864 (i.e., a ligand of TSPO) increases the low level of neuroactive steroids, such as PREG, PROG and DHT, assessed in the sciatic nerve of STZ-animals [115]. In addition, it prevents the impairment of NCV and thermal threshold, while also restoring skin innervation density and P0 mRNA levels, and improving Na^+^, K^+^-ATPase activity [115]. Altogether these observations suggest that activation of LXR or TSPO, by increasing the levels of neuroactive steroids in the sciatic nerve, exerts neuroprotective effects in diabetic neuropathy and consequently may represent an alternative to the direct treatment with neuroactive steroids.

### 4.2. Aging Process

Aging induces significant biochemical and morphological changes in the peripheral nerves. Indeed, aging is associated with a decrease of PMP22 and P0 synthesis, and atrophy of large myelinated fibers, while myelin sheaths undergo an increase in thickness and show various irregularities, such as myelin ballooning, reduplication and remyelination [7,17]. These alterations are counteracted by neuroactive steroid treatment. In particular, the low levels of P0 present in aged male rats are increased by the treatment with PROG or DHP, while those of PMP22 are significantly increased by THP treatment [7,17].

PROG and its metabolites also have a clear beneficial effect on the number and shape of myelinated fibers as well as on the frequency of myelin abnormalities [7,17]. In particular, the most evident effect of these molecules is the significant increase in the number of myelinated fibers of small caliber (<5 µm). This positive effect on the number of myelinated fibers may reflect an increased remyelination of small fibers in the sciatic nerve of aged rats. In addition, treatment with PROG, DHP or THP has also shown an important effect on myelin abnormalities of axons by reducing the frequency and the proportion of nerve fibers with irregular shapes. These effects seem to be a peculiarity of PROG and its metabolites since neither T nor DHT or 3α-diol are able to influence the morphological parameters reported above [7,17].

### 4.3. Chemotherapy-Induced Peripheral Neuropathy

Chemotherapy-induced peripheral neuropathy (CIPN) is one of the most common side effects of patients undergoing treatment for cancer. Clinically, CIPN shows deficits in sensory, motor, and autonomic functions. Several classical chemotherapeutics, such as platinum derivatives, vinka alkaloids and taxanes, are well-established causes of CIPN [116,117,118]. 

On the basis of the neuroprotective effects exerted by neuroactive steroids in other experimental models of PN, their potential effects have also been evaluated in a model of CIPN, such as that induced by docetaxel (i.e., a semisynthetic taxane employed as an antineoplastic agent for the treatment of breast, ovarian, and non-small cell lung cancer) [119]. Results obtained so far have shown that PROG as well as its metabolite DHP have positive effects against docetaxel-induced neuropathy, improving thermal threshold, preventing NCV changes and degeneration of skin nerves in the footpad [119]. Furthermore, neuroactive steroid treatment also prevents the changes in gene expression of P0, PMP22 and MBP induced by docetaxel in the sciatic nerve of male rats [119]. Similarly, PROG metabolites (i.e., DHP and THP) counteract the decrease in the number of intra-epidermal nerve fibers, of NCV and the pain transmission abnormalities induced by vincristine [120].

### 4.4. Physical Injury

Peripheral nerves are often exposed to physical damage (i.e., cutting, crushing, entrapment with nerve compression) and this may induce a kind of PN classifiable according to the severity of nerve injury. In the case of crush or cut of the nerve, a process known as Wallerian degeneration occurs. Thus, the part of axon distal to the site of injury degenerates, with fragmentation of myelin and involvement of Schwann cells and macrophages [121,122].

The protective and regenerative effects of neuroactive steroids have been demonstrated in experimental models of degeneration occurring after physical injury of peripheral nerves. Indeed, the gene expression of P0 is increased by neuroactive steroids after nerve transection. In particular, PROG or DHP treatment significantly increase the low messenger levels of P0 in the distal portion from the cut of the rat sciatic nerve [21]. In addition, the same steroids counteract the alterations observed after crush-induced degeneration. For example, increased myelin protein expression and restored activity of the Na^+^, K^+^-ATPase pump, coupled to improved nociception, have been observed in nerve crushed animals treated with PROG or DHP [123]. Important results have been also obtained with other neuroactive steroids. For instance, T or DHT treatment accelerates regeneration and functional recovery of nerves in many rodent peripheral nerve injury models (e.g., hamster facial motoneuron, rat sciatic motoneuron, rat pudendal motoneuron) [26,124,125,126,127]. Finally, it has been reported that DHEA improves axonal regeneration after sciatic nerve transection [128], as well as after crush injury in rats [129].

## 5. Neuroactive Steroids, Sex and Peripheral Neuropathy

**Figure 2:**
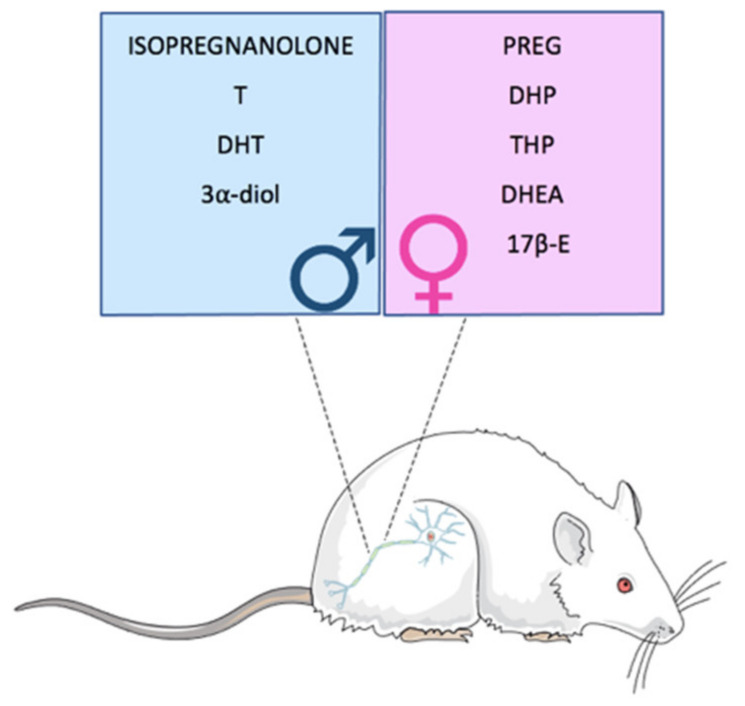
Sexual dimorphism of neuroactive steroid levels in peripheral nerves. Neuroactive steroid levels in rat sciatic nerve show sexual dimorphism. Further details are described in the text. T: testosterone; PREG: pregnanolone; 17β-E: 17β-estradiol.

In physiological conditions, neuroactive steroid levels present sex differences, not only as expected in plasma levels, but also in different brain areas, in the cerebrospinal fluid (CSF), and in peripheral nerves (e.g., the sciatic nerve). For instance, the levels of PREG are higher in the CSF, cerebral cortex, hippocampus and cerebellum of male rats, with respect to females, while the same neuroactive steroid shows elevated levels in the spinal cord and plasma of females in the diestrous phase [35]. In addition, in the sciatic nerve, neuroactive steroid levels showed sex differences (Figure 2). In particular, levels of PREG, DHP, THP, DHEA and 17β-estradiol are higher in the sciatic nerve of females than in the males while the levels of isopregnanolone, T, DHT and 3α-diol are higher in the male sciatic nerve [35].

Neurosteroidogenesis shows sex-dimorphic features [130] and it may be also influenced by the peripheral sex steroid environment [131]. Indeed, in the CNS, gonadectomy alters the levels of neuroactive steroids depending on the brain areas considered [131]. In addition, changes occurring in the CNS are different to what is observed in the PNS and both do not reflect the changes occurring in plasma [131], supporting the concept of neurosteroidogenesis. In addition, the effects of gonadectomy depend on sex and on the duration of gonadal hormone deprivation (i.e., long- vs. short-term castration) [131]. For instance, it has been observed that long-term (4 months) gonadectomy induces an increase of PREG levels in male rat sciatic nerve compared to control values, but this effect is not observed after short-term (7 days) gonadectomy, where, in contrast, a decrease of the levels of this neuroactive steroid occurs. Interestingly, the levels of PROG and its metabolite, THP, in rat sciatic nerve respond to gonadectomy in a sex-dimorphic way. Indeed, only in females the levels of these neuroactive steroids are decreased by long-term gonadectomy. On the contrary, the levels of DHEA, are increased by long-term, but not by short-term, gonadectomy in male animals, while they are not affected in females. Further metabolites of DHEA, such as T and DHT, are significantly decreased by short- and long-term gonadectomy in males but not in females [131].

As mentioned above, PROG and its metabolites regulate myelination. Interestingly, the regulation of the PMP22 and P0 expression is sexually dimorphic. Indeed, PROG and DHP stimulate P0 mRNA levels in cultures of male but not in female Schwann cells, while THP is only effective in Schwann cells derived from females. Moreover, PMP22 is stimulated by PROG only in male Schwann cells while THP is effective only in female Schwann cells [132]. Sex dimorphic effects have also been reported in Schwann cell proliferation. In particular, it has been observed that PROG stimulates Schwann cell proliferation only in female cultures [25].

Sex differences have been reported in the incidence and/or manifestation of numerous disorders of PNS. In agreement, the levels of neuroactive steroids also show sex differences under pathological conditions [84,133]. For instance, diabetic peripheral neuropathy is more frequent in men than in women (ratio male/female 2.9) [134,135] and men develop neuropathy approximately 4 years earlier than women [136]. Moreover, other sex differences are related to motor nerve conduction and atrophy, which are more common in males. On the contrary, in females, the major symptoms are related with pain and negative sensory signs [137,138]. Sex differences have been also reported in nerve regeneration after sciatic nerve injury in healthy rats as well as in an experimental model of type 2 diabetes, such as Goto-Kakizaki rats [139]. 

As mentioned above, in the STZ rat model, neuroactive steroid levels observed in peripheral nerves are affected by the pathology. These alterations are also sex-dimorphic [38]. Indeed, long-term diabetes (i.e., three months after the induction) affects the levels of PREG, T, DHT and 3α-diol in the male rat sciatic nerve, while an alteration of PROG, THP and isopregnanolone levels is observed in the female sciatic nerve [38]. Interestingly, neuroactive steroid levels in the sciatic nerve are also altered after one month from diabetes induction. In particular, in male STZ rats, a reduction in T and DHT levels with an increase of THP levels were observed. In female sciatic nerves, the levels of the neuroactive steroids assessed were unaffected [110]. In the same experimental model, it has been observed that gonadectomy protects female, but not male animals, from diabetic peripheral neuropathy by increasing the local levels of specific neuroactive steroids, such as DHEA, T and DHT [104]. In agreement, DHEA treatment protects gonadally intact STZ-female, but not male animals, from diabetic peripheral neuropathy [91].

In experimental short-term diabetes, axonal transport and mitochondrial functions (i.e., the organelle where the limiting step of steroidogenesis is located) are also affected in a sex dimorphic way [110]. Indeed, gene expression and axonal protein content of kinesin family members, like KIF1A, KIF5B, KIF5A and Miosin Va, involved in axonal transport, are only affected in male STZ-rats. Similarly, the expression of respiratory chain complex IV as well as key regulators of mitochondrial dynamics (e.g., fission/fusion processes) are affected only in diabetic male rats [110]. 

Sex-dimorphic alterations of neuroactive steroid levels have been reported also in other experimental models of PN. Indeed, as demonstrated in the sciatic nerve of male and female PMP22 transgenic rats (i.e., an experimental model of Charcot–Marie–Tooth type 1), the levels of 3α-diol are strongly decreased only in males and those of isopregnanolone only in females [37]. In addition, it has been recently demonstrated that the latencies of potentials, a feature of PN occurring in Gaucher disease (i.e., a rare lysosomal storage sphingolipidosis caused by mutation in the *GBA1* gene) show a significant association with sex steroids (i.e., T) only in female patients [140]. Finally, sex differences have been also reported in PN associated with antiretroviral therapy, showing a higher incidence in female patients [141].

## 6. Neuropathic Pain and Neuroactive Steroids

Neuropathic pain, an important component of PN, is a chronic pain that may arise after an injury or disease affecting the somatosensory system [142] and that is considered a maladaptive response of the nervous system to damage [143]. Etiological factors producing neuropathic pain include trigeminal neuralgia, postherpetic neuralgia, painful radiculopathies, diabetic polyneuropathies, central pain produced by stroke or spinal cord injury (SCI). Moreover, traumatic postsurgical and CIPN also represent common conditions [142,144]. Patients show common clinical characteristics, with the presence of both positive and negative symptoms, representing gain and loss of function of the somatosensory pathway, respectively. Positive phenomena include both ongoing (also called spontaneous) and evoked pain, while negative phenomena arise as sensory loss [142].

Unfortunately, even if neuropathic pain afflicts 7–10% of the general population worldwide, the treatment of this condition is still unsatisfactory, with a substantial number of patients having decreased quality of life with little or no benefit from available treatment [145]. 

As mentioned above, when the somatosensory system is injured or damaged by a disease, the painful signals from PNS to the cortex can be altered, resulting in a maladaptive process [146]. This maladaptive process may affect both the PNS and CNS, inducing an increased excitability of the neuronal pain processing circuitry and generating peripheral or central sensitization, symptomatically expressed as hyperalgesia and allodynia [147]. These events involve different mechanisms, such as: i) changes in the phenotype of sensory neurons, affecting different molecules and in particular neuropeptides like calcitonin gene-related peptide and substance P (SP); ii) imbalance of excitatory and inhibitory neurotransmission at the spinal cord dorsal horn (DH) level, involving decreased GABA_A_ receptor activity and increased NMDA receptor-mediated currents; iii) an exacerbated neuroimmune reaction, with glial activation and the production of inflammatory mediators [143,148,149,150]. 

As mentioned above, the treatment of neuropathic pain is only partially effective and for this reason, it is necessary to propose new strategies, such as suppressing symptoms or preventing the different events leading to maladaptive plasticity.

**Figure 3:**
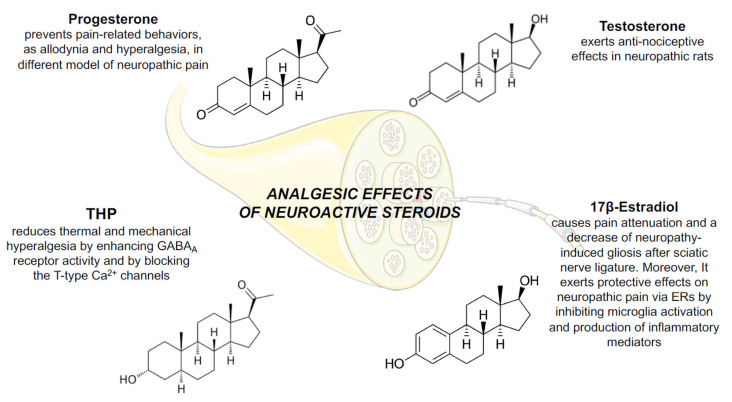
Analgesic effects of neuroactive steroids in the peripheral nervous system. Analgesic effects of progesterone, testosterone, THP and 17β-estradiol in several neuropathic pain experimental models. Abbreviations and details are reported in the text.

As extensively demonstrated, neuroactive steroids control important aspects of development, activity and plasticity of the nervous system [5,84,151,152,153,154], therefore they appear as interesting candidates to manage neuropathic pain. Figure 3 summarizes the main effects exerted by neuroactive steroids in managing neuropathic pain, as detailed below.

Among steroidal compounds, glucocorticoids, like dexamethasone and methylprednisolone, are routinely used to reduced pain, since they are able to inhibit the pro-inflammatory cytokines and to up-regulate lipocortin (annexin-1) (i.e., a protein that suppresses phospholipase A2, blocking eicosanoid production, and inhibiting various leucocyte inflammatory events) [155]. However, as reported in experimental models of neuropathic pain, glucocorticoid receptors (GR) are upregulated in the spinal DH and contribute to hyperalgesia [156]. In addition, neuropathic pain is exacerbated by stress via glucocorticoid and NMDA receptor activation [157]. Moreover, in a chronic constriction injury (CCI) model, glucocorticoid activation in the early phase of pain induction, produced hyperalgesia. Finally, in the same experimental model, a GR antagonist mediated a slight mechanical hypoalgesia, mediated by NMDA receptors, rather than acting on inflammatory mediators [158]. Thus, concern in the use of glucocorticoid for pain treatment has been raised, and a possible role for GR in maladaptive plasticity has been proposed [159]. 

In addition to glucocorticoids, PROG and its metabolites are also known to reduce pain-related behaviors in different models of neuropathic pain [101,120,160,161,162,163,164,165,166,167].

As mentioned above, PROG and its metabolites are able to restore biochemical, functional and morphological parameters after peripheral nerve injury [21,123]. For example, in the CCI model, 14 days of PROG treatment, starting 12 days after CCI, improves the electrophysiological changes in motor and sensory conduction velocity, thermal hyperalgesia, and mechanical allodynia produced by injury [168]. Moreover, Coronel and colleagues reported that early PROG administration, when it is able to block the post-SCI inflammatory cascade, reduces the number of glial fibrillary acidic protein (GFAP) positive cells and avoids the development of mechanical and thermal allodynia [162]. In a different model, sciatic nerve ligature, the same authors reported that PROG administration protects from developing mechanical allodynia and promotes a better response to cold stimulation, through a reduction of spinal cord central pain sensitization mediators (i.e., the NMDA receptor subunit NR1 and the gamma isoform of protein kinase C—PKC) [160,161].

Similarly, THP plays an important role in pain modulation. As mentioned above, this steroid is a positive allosteric modulator of GABA_A_ receptors [169] and, indeed, probably by this mechanism, it reduces thermal and mechanical hyperalgesia after sciatic nerve ligature [170]. 

In experimental models of painful CIPN, PROG, as well as DHP and THP, are able to stop neuropathic symptoms evoked by antitumoral drugs like oxaliplatin [163] or vincristine [120]. Recently, it has been shown that the prophylactic administration (i.e., three injections every 4 days before and during the chemotherapy cycle) of 17α-hydroxyprogesterone caproate, a synthetic derivate of PROG, is able to prevent allodynia and the mechanical and cold hypersensitivity caused by oxaliplatin. In addition, the early administration of this steroid prevents chemotherapy-triggered neurotoxicity and glial activation, suggesting a promising therapeutic strategy [171].

Many studies have also shown an important role of a heterogeneous family of voltage-gated Ca^2+^ channels in the development of neuropathic pain [172,173]. Therefore, the pharmacological modulation of T-type Ca^2+^ channel currents may be of value in the management of this type of pain [174,175]. Interestingly, it has been reported that an important part of the anti-nociceptive action of THP in neuropathic pain is due to its ability to block T-type Ca^2+^ channels [176]. This hypothesis is supported by the fact that structural modifications of 3α,5α-reduced neuroactive steroids, which eliminate the interaction with calcium channels but preserve that on GABA_A_ receptors, cause complete loss of analgesic activity induced by these steroids. These observations suggest that their peripheral analgesic action is mediated mainly by T-channels and only to a smaller extent by GABA_A_ receptors [176]. Indeed, administration of a synthetic 3α,5α-reduced analog of neuroactive steroids, such as (3β,5α,17β)-17-hydroxyestrane-3-carbonitrile (ECN, i.e., a blocker of T-channels without potentiating effects on GABA_A_ receptors) induces potent analgesia in healthy rats. Interestingly, when this treatment is combined with a GABA_A_ selective neurosteroid without analgesic effect per se, such as (3α,5α)-3-hydroxy-13,24-cyclo-18,21-dinorchol-22-en-24-ol (CDNC24), the antinociceptive effects of ECN are strongly potentiated. This synergistic effect is abrogated by bicuculline, an antagonist of GABA_A_ receptors. Based on these results, a correlation between GABA_A_ receptors and T-channels in peripheral nociceptive signaling has been suggested, and this cooperation might be used in order to alleviate acute pain [176]. In addition, these two synthetic analogs (i.e., ECN and CDNC24) may also alleviate pain in chronic neuropathy. For instance, in a model of CCI of the sciatic nerve, local intraplantar injection of either CDNC24 or ECN, is more effective in alleviating thermal nociception in neuropathic animals than THP [165]. This can be explained by the fact that synthetic steroids, in comparison to THP, are more selective to either GABA_A_ and/or T-channels [165].

As mentioned above, after a lesion or a disease of the nervous system, PROG exerts beneficial effects while avoiding maladaptive plasticity and pain behaviors. These effects may be mediated by the capability of PROG to interact with a variety of receptors, such as PR, mPR, and PGRMC1. In addition, PROG acts as a competitive antagonist of sigma-1 receptor [52], expressed in the dorsal spinal cord and associated with pain and central sensitization [177]. On the other hand, the anti-allodynic and anxiolytic effects of PROG may be also due to its rapid conversion into THP, which reinforces inhibitory neurotransmission by acting as a positive allosteric modulator of GABA_A_ receptors [169] and also enhancing expression of specific receptor subunits [178]. In particular, in pain perception, the correlation among GABA_A_ receptors, neuroactive steroids and the PKC isoform epsilon has also emerged. For instance, it has been proposed that 3α,5α-reduced neuroactive steroids may take part in a compensatory mechanism in response to continued activation of the spinal nociceptive system during pain conditions. In contrast, PKC epsilon prevents further 3α,5α-metabolite-dependent potentiation, without decreasing the basal modulatory effect of these neuroactive steroids [179,180]. Recently, this concept has been further extended by proposing a mechanism involving THP, brain-derived neurotrophic factor and its receptor trkB, and PKC epsilon in neuropathic pain [181].

Neuroactive steroids, in order to control pain in the spinal cord, may also be locally synthesized. Indeed, different studies have demonstrated that the spinal cord DH of rats expresses many key steroid-synthesizing enzymes [29,182,183,184,185,186,187,188]. For example, in a rat experimental model of neuropathic pain obtained by sciatic nerve ligature, an up-regulation of enzymatic pathways (P450scc and 3α-HSOR) in DH resulted in increased synthesis of THP [170,184,186]. 

THP synthesis may be also regulated by SP, the major nociceptive neuropeptide released by primary afferents. Indeed, this nociceptive neuropeptide, through THP biosynthesis inhibition in the spinal cord, may indirectly decrease the spinal inhibitory tone, thus facilitating noxious signal transmission [189]. 

The central role of P450scc induction in neuropathic pain has been assessed in a model of CCI. Sciatic nerve injury, during the induction phase of neuropathic pain (i.e., 0–3 days after CCI), significantly increases the spinal P450scc expression in astrocytes but not in neurons [190]. The early block of P450scc, through the administration of a P450scc inhibitor, significantly suppresses the development of neuropathic pain after CCI. The authors of this study proposed that the effect could be mediated by inactivation of astrocyte serine racemase, the enzyme converting L-serine to D-serine, which is a co-activator of NMDA receptors. Indeed, the production of D-serine potentiates NMDA receptor-mediated signaling, contributing to the development of mechanical allodynia following peripheral injury [190]. These observations suggest a possible therapeutic use of P450scc inhibitors in order to modulate the development of neuropathic pain. P450scc, as well as P450c17, the enzyme converting PREG into DHEA, plays an important role in the development of neuropathic pain. Indeed, in the CCI model, the expression of this enzyme is significantly increased in spinal astrocytes [191]. During the induction phase of neuropathic pain in the CCI model, the administration of ketoconazole, a P450c17 inhibitor, reduces the development of allodynia and thermal hyperalgesia in the ipsilateral hind paw [191]. Based on these data, it has been suggested that the development of mechanical allodynia is associated with P450c17 activity during the induction phase of neuropathic pain. In agreement, the intrathecal administration of DHEA produced a rapid pro-nociceptive and a delayed anti-nociceptive action in the sciatic nerve ligature model. These observations suggest that DHEA itself induces a rapid pro-nociceptive action while, after its metabolism into androgens, it exerts analgesic effects. In agreement, intrathecal T administration promotes anti-nociceptive behaviors in the same experimental model [183]. Therefore, another possible therapeutic strategy may rely on the modulation of the P450c17 enzyme.

Another important component of neurosteroidogenesis, like TSPO, seems to be involved in the modulation of neuropathic pain. For instance, TSPO expression is upregulated in astrocytes and microglial cells in spinal cord DH after L5 spinal nerve ligation [192]. Intrathecal administration of a TSPO agonist, such as Ro5-4864, results in decreased neuropathic pain mediated by an inhibition of activated astrocytes. These results suggest that the inhibition of spinal astrocytes, involved in neuropathic pain establishment [193,194,195], exerts a role in the protective effects of Ro5-4864. Pretreatment with the steroidogenic inhibitor aminoglutethimide abolishes the anti-nociceptive action of this TSPO ligand, further confirming that the protective effects observed are due to activation of neurosteroidogenesis. Indeed, protective effects of TSPO ligands against neuropathic pain linked to the increase of neurosteroidogenesis have also been reported in a different experimental model of PN [115]. Therefore, novel therapeutic approaches against neuropathic pain could take ligands for upregulated proteins, such as TSPO, into consideration. 

At the base of the pain perception, there is a possible influence of sex. In general, women report more pain than men and exhibit higher risk for developing chronic pain [196,197]. In addition, as demonstrated in cohorts of patients with type 2 [198] or type 1 diabetes [199,200], females show higher frequency and intensity of neuropathic pain. In agreement, sex differences have also been reported in experimental models of different PNs. For instance, in a mouse model of paclitaxel-induced neuropathic pain, cold allodynia is more evident in females than in male mice [201]. In an experimental model of HIV-1-associated neuropathic pain, increased cold sensitivity and mechanical allodynia have been reported to be higher in female than in male mice [202]. Interestingly, ovariectomy produces neuropathic pain-like behaviors more similar to male mice in comparison to sham females [202], supporting a role for sex steroids in pain perception. After sciatic nerve ligature, male mice report a gradual decrease of allodynia and a complete recovery, while in female animals, allodynia is still present after four months of neuropathy induction [203]. 

Several clinical studies show that gonadal steroids have a central role in gender differences in pain and analgesia. In addition, a possible correlation may exist between alterations in neuroactive steroid levels, expression of their receptors and the development of chronic pain [204,205]. Indeed, while androgens, in particular T levels, are higher in males and exert analgesic effects in human and experimental models, estrogens show both hyperalgesic and analgesic effects depending on the experimental conditions [204,206,207,208]. 

The role of peripheral sex steroid hormones on endogenous pain control has been assessed in an experimental model of pain (i.e., formalin injection) comparing the response between intact and gonadectomized rats [209,210]. In both acute (i.e., phasic noxious stimuli) and tonic (i.e., continuous noxious stimuli) nociceptive phases, gonadectomized male rats present more pain than intact male rats while gonadectomized females exhibited less nociceptive responses than the control group but only in the inhibitory interphase (i.e., phase related to active inhibitory mechanism rather than an absence of nociceptive activity) [209]. Hormonal supplementation in this experimental model has shown that 17β-estradiol and PROG mainly act on the inhibitory mechanism of pain while T plays a protective role in pain perception [210].

In the paclitaxel CIPN model, it has been shown that female sex steroid hormones (i.e., PROG and 17β-estradiol) are involved in modifying pain intensity, by proinflammatory cytokines in the sensory nerves [211]. Promising results have been obtained with 17β-estradiol treatment after sciatic nerve ligature. Indeed, this steroid induces pain attenuation, a functional improvement of the injured limb, a faster regenerative process of the sciatic nerve as well as a decrease of neuropathy-induced gliosis [203]. 

17β-estradiol exerts analgesic effects via ERs expressed in L4-L5 DH neurons. For instance, in a neuropathic pain model of SCI, 17β-estradiol treatment inhibits microglia and astrocyte activation. In particular, the factors known to be activated in astrocytes and microglia, like JNK, p38MAPK and ERK, present decreased levels as a consequence of 17β-estradiol treatment and, this, in turn, reduces the expression of pro-inflammatory mediators, such as IL-1β, IL-6 and Cox-2 [212]. These data highlight the protective effects of 17β-estradiol on neuropathic pain mediated by ER. In addition, ER-β selective agonists, as well as other structural analogues, present anti-allodynic effects in the spinal nerve ligation [213] and CIPN pain models [214], supporting the involvement of ER-β in the analgesic effects mediated by 17β-estradiol.

Altogether, these observations support the idea that the fluctuations of sex steroid levels may significantly influence pain perception and may provide an interesting background for future investigations aimed to develop effective sex-specific steroid-based strategies against pain.

## 7. Conclusions

Data here reported support the concept that neuroactive steroids, synthetic ligands acting on their receptors or inducing their synthesis, may improve PN symptoms, including neuropathic pain and consequently may represent an interesting possible therapeutic strategy. In addition, based on the sexual dimorphism of neuroactive steroids as well as of PN here discussed, a gender specific treatment based on these compounds may be also proposed. Therefore, even if the molecular mechanisms behind PN need to still be investigated in detail, the pleiotropic effects exerted by neuroactive steroids represent an interesting therapeutic option that deserves deeper exploration.

## Abbreviations

3α-diol	5α-androstane-3α,17β-diol
3α-HSOR	3α- hydroxysteroid oxidoreductase
3β-diol	5α-androstane-3β,17β-diol
3β-HSD	3β-hydroxysteroid dehydrogenase
3β-HSOR	3β-hydroxysteroid oxidoreductase
5-HT	Serotonin
5α-R	5α-reductase
AMPA	α-amino-3-hydroxy-5-methyl-4-isoxazolepropionic acid
AR	Androgen receptor
ARO	Aromatase
CCI	Chronic constriction injury
CDNC24	(3α,5α)-3-hydroxy-13,24-cyclo-18,21-dinorchol-22-en-24-ol
CIPN	Chemotherapy-induced peripheral neuropathy
CNS	Central nervous system
CSF	Cerebrospinal fluid
DH	Dorsal horn
DHEA	Dehydroepiandrosterone
DHP	Dihydroprogesterone
DHT	Dihydrotestosterone
DRG	Dorsal root ganglia
ECN	(3β,5α,17β)-17-hydroxyestrane-3-carbonitrile
ER	estrogen receptor
GABA	γ-amino butyric acid
GFAP	glial fibrillary acidic protein
GR	glucocorticoid receptor
IL	interleukine
LXR	Liver X Receptor
MBP	Myelin basic protein
mPRs	Membrane progesterone receptors
NCV	Nerve conduction velocity
NMDA	N-metyl-D-aspartate
P0	Glycoprotein zero
P450scc	Cytochrome P450 side chain cleavage
PGRMC1	Progesterone receptor membrane component 1
PKC	Protein kinase C
PMP22	Peripheral myelin protein
PN	Peripheral neuropathy
PNS	Peripheral nervous system
PR	Progesterone receptor
PREG	Pregnenolone
PROG	Progesterone
SCI	Spinal cord injury
SP	Substance P
SRC-1	Steroid receptor coactivator-1
SREBP-1c	Sterol regulatory element binding protein
StAR	Steroidogenic Acute Regulatory Protein
STZ	Streptozotocin
T	Testosterone
THP	Tetrahydroprogesterone
TSPO	Translocator protein-18kDa
